# Supplementary material for: Learning curve and surgical outcome of robotic assisted colorectal surgery with ERAS program
Source: Sci Rep. 2022 Nov 29;12:20566. doi: 10.1038/s41598-022-24665-w (PMC9709162; doi:10.1038/s41598-022-24665-w)
Supplement: Supplementary file 1 — Supplementary Information 1. [file 41598_2022_24665_MOESM1_ESM.zip › LSC Raw data-Ñ[▒K/064/H_ñw╝╨░O▒Kñσ.pdf]

正式摘要(電子簽章：000000125808696)

W86 / W86 ☐英文標題(注意:無英文姓名-請補病人基本檔!) ☐【讀取】憑證

臺中榮民總醫院

出院病歷摘要

[redacted]

(8)流水編號	
(9)入院日期	109/02/04 (2020) 直腸外科 W86 - 193
(10)轉科日期	
(11)出院日期	109/02/14 (2020) 住院天數計 10 日

診斷

- 入院:Ca of rectum with liver metastasis
- 出院:Ca of rectum with liver metastasis s/p LAR on 2020/02/06

主訴

- bloody stool for period of time

病史

- This is a 75 year old female patient with history of

  1. Type 2 Diabetes

  2. appendicitis s/p

  3. Parkinsonism under madopar

  According to the patient, she suffered from bloody stool recently. And both frequency and amount had increased. She recieved colonoscopy in outside hospital and rectal tumor was noted. She came to our CRS hospital for 2nd opinion and was admitted for schedule surgery.

體檢發現

- Anal inspection:
  Recto digital examination:
  Sigmoidoscopy:
  Anoscopy:
  Abdomen:
  - General appearance :
    - easy looking , conscious clear , without cardiopulmonary distress
    - Vital signs自動帶入date 體溫(BT) 脈搏(P) 呼吸(R) 血壓(BP) 身高(BH) 體重(BW) BMI
      20200204 36.8℃ 92次/分 17次/分 167/89mmHg 146cm 55.5kg 26.0
  - HEENT :
    - Eyes: no anemic conjunctiva, no icteric sclera, normal EOM
    - ENT: no otorrhea, no rhinorrhea, no erythematous mucosa, no injected tonsil, no deformity
  - Neck: supple, freely movable, trachea midline,no deformity, no engorged jugular vein , no palpable thyroid or lymph node enlargement
  - Chest:
    - Inspection: Grossly normal, symmetric and fully expansion, no accessory muscle used, no operation scar, no spider angiomata, no gynecomastia
    - Palpation: no palpable mass or nodule, no subcutaneous emphysema, no tenderness
    - Ausculation: clear breathing sound
  - Heart:
    - Percussion: normal heart size
    - Ausculation: regular heart beat, normal S1, S2, no gallop, no murmur
  - Abdomen:
    - Inspection: in
      - flat contour, symetrically, no scar, no stria, no dilated veins, no rashes
    - Ausculation: normoactive bowel sound, no abdominal bruits
    - palpation: soft, no muscle guarding or rigidity, no local tenderness, no rebounding pain, no palpable mass, no palpable pulsation
    - Percussion: no shifting dulllness, no hepatomegaly, no splenomagaly
  -

Peripheral pulsation :	CA	BA	RA	FA	PA	PTA	DPA
Right side :	++	++	++	++	++	++	++/+++
Left side :	++	++	++	++	++	++	++/+++

  - Genito-anus: no tenderness or palpable mass lesion or rectal shelf, normal external genitalia, normal anal tone, brown stool

  CRS CONDITION

  -
    - Lung

手術日期及方法

- 手術日期及方法20200206 1.Laparoscopic low anterior resection with end-to-end anastomosis, double stapling, EndoGIA 45/4.1mm*2, CDH 29mm, bilateral doughnuts: intact, air leak test: done with air leak.
  2. High ligation (+) above the left colic artery.
  3. Cut margin: proximal: 15 cm, distal: 3 cm.
  4. Take down of splenic flexure: nil
  5. Drain: a 12mm penrose drain in pelvis
  6. Repair of mesentery defect:nil

住院治療經過

- After the admission, pre op survey was done which showed liver metastasis. GS doctor was consult and combine surgery was arranged on 2020/02/06. However, due to her condition, she only recieved LAR with anastomosis. After days of recovery, the patient was discharge on 2020/02/14 under relative stable condition.

合併症

- nil

一般檢查

- 累積報告 :

DATE	TIME	ALB	TP	BIL.T	BIL.D	ALKP	AST	ALT	LDH	CHOL	TG	HDL-C	LDL	UA	NH3	AMY
1090205	1835	3.8	6.9			92	42	19		259	83	56	178	5.6		
1090206	1935	3.3		0.3			33	19								
1090209	0735			0.5		55		10	436							

DATE	TIME	WBC	RBC	HGB	HCT	MCV	PLT	BLST	NEUT	BAND	SEG	LYM	MON	EOS	BAS
1090205	1834	4680	3.94	12.5	38.2	97.0	168		59.2			30.8	7.9	1.5	0.6
1090206	1936	6580	3.58	11.1	34.5	96.4	146		77.9			16.1	5.6	0.2	0.2

1090209	0722	8080		9.6	30.4		125		78.2			12.4	8.7	0.6	0.1

DATE	TIME	NA	K	CL	CA	BUN	CREAT	Mg	P	CRP	ESR	PCT	LIPASE	LACTATE
1090205	1835					15	0.83			0.461				
1090206	1935	143	2.9			12	0.65							
1090209	0731													9.5
1090209	0735	138	3.3		7.5	9	0.65							

DATE	TIME	GLUCOSE	PAB	TRF	ZN	CU	KETONE	CK	CKMB	PT-P	PT-C	APTT-P	APTT-C	TRO-T
1090205	1835	70												

DATE	TIME	PH	BEB	SCVO2	PO2	HCO3	HGB	PCO2	TCO2
1090209	0720	7.439	-1.5	94.4	69.8	22.1		33.4	23.1

- 放射線報告 :檢驗項目 **:CT ABDOMEN-SURVEY** 報告日期 **:1090123**

主　訴：
Result:
Finding:
Imaging Report Form for Colorectal Carcinoma (AJCC 8th)
1. Imaging Date: 20200120
Imaging Modality, Imaging by
CT scan
Imaging Protocol:
Post contrast CT of abdomen showed:
2. Tumor location / Size
Location:
Rectum
Size:
Non-measurable
3. Tumor invasion
Tumor invades non-peritonealized pericolonic or perirectal tissues
4. Regional nodal metastasis
N1: Yes
number of suspicious lymph node: 2
location:
Pericolic/perirectal
5. Distant metastasis (In this study)
Two or more organs
6. Other findings:
* Mucosal thickening over rectum, axi#67-72, cor#27-34, rectal ca is considered. suggest further evaluation.
* several small lymph node about 1cm in diameter over presacral region(axi#65),
* A 1.3cm right paracaval lymph node(axi#29) and two 1.6cm hepatic hilar lymph nodes(axi#23-24) are also noted, metastatic lymphadenopathy is considered.
* Multiple poor enhancing foci over bilateral lobes of liver, the largest one about 3.4cm in diameter over S4 of liver, hepatic metastasis is considered.
> A poor enhancing lesion over lower pole of right kidney, in favor of renal cyst, suggest follow up.
> No space occupying lesion over gallbladder, pancreas, spleen, bilateral adrenal gland and left kidney
> A soft tissue density with calcified spot over left adnexal region, axi#64, suggest correlation with GYN condition.
> Bil. clear basal lung
> DJD change of T-L spines with marginal spurs formation, s/p vertebroplasty over L1
Impression:
1. Recal ca, T3N1M1b, with regional and metastatic lymphadenopathy of pericolic, retroperitoneal hepatic hilum, also with hepatic metastasis. Suggest further management.
2. Others, As above description.

特殊檢查

病理報告

其它

出院時情況

- 3.改門診治療

出院指示

藥名	劑量	劑量單位	用法	頻次	數量	數量單位	現況	天數	單位
CefaDROxil-500 cap	1	CAP	PO	Q12H	6	CAP	TH	3	天
$Biofermin tab	2	TAB	PO	TID	18	TAB	TH	3	天
Ulstop tab 20mg	1	TAB	PO	BID	6	TAB	TH	3	天
Lactam tab 500mg	1	TAB	PO	Q6HPRN	12	TAB	TH	3	天

出院計劃

主治醫師_2062F 蔣鋒帆_ / 住院醫師_2328K 邱俊凱_

正式摘要**(電子簽章：000000185808696)**

W86 / W86

doctorRank:B
